# Budding Yeast Pch2, a Widely Conserved Meiotic Protein, Is Involved in the Initiation of Meiotic Recombination

**DOI:** 10.1371/journal.pone.0039724

**Published:** 2012-06-22

**Authors:** Sarah Farmer, Eun-Jin Erica Hong, Wing-Kit Leung, Bilge Argunhan, Yaroslav Terentyev, Neil Humphryes, Hiroshi Toyoizumi, Hideo Tsubouchi

**Affiliations:** 1 MRC Genome Damage and Stability Centre, University of Sussex, Brighton, United Kingdom; 2 Life Sciences, University of Sussex, Brighton, United Kingdom; 3 Graduate School of Accounting, Waseda University, Tokyo, Japan; 4 Department of Applied Mathematics, Waseda University, Tokyo, Japan; 5 ImmunoGen, Inc., Waltham, Massachusetts, United States of America; National Cancer Institute, United States of America

## Abstract

Budding yeast Pch2 protein is a widely conserved meiosis-specific protein whose role is implicated in the control of formation and displacement of meiotic crossover events. In contrast to previous studies where the function of Pch2 was implicated in the steps after meiotic double-strand breaks (DSBs) are formed, we present evidence that Pch2 is involved in meiotic DSB formation, the initiation step of meiotic recombination. The reduction of DSB formation caused by the *pch2* mutation is most prominent in the *sae2* mutant background, whereas the impact remains mild in the *rad51 dmc1* double mutant background. The DSB reduction is further pronounced when *pch2* is combined with a hypomorphic allele of *SPO11*. Interestingly, the level of DSB reduction is highly variable between chromosomes, with minimal impact on small chromosomes VI and III. We propose a model in which Pch2 ensures efficient formation of meiotic DSBs which is necessary for igniting the subsequent meiotic checkpoint responses that lead to proper differentiation of meiotic recombinants.

## Introduction

Meiosis plays a central role in sexually reproducing organisms by producing haploid gametes from diploid parental cells [1]. During meiosis, a single round of DNA replication is followed by two successive rounds of nuclear division, meiosis I and meiosis II respectively. Homologous chromosomes segregate in meiosis I whereas, in meiosis II, sister chromatids are separated like mitosis.

During prophase of meiosis I, homologous recombination is highly induced and plays two essential roles [2]. First, recombination ensures that each chromosome finds its homologous partner. Second, a subset of recombination events are resolved as crossovers, establishing physical connections between homologs. These crossovers are essential for ensuring the proper alignment of chromosomes on the spindle apparatus, and thus their faithful segregation at meiosis I.

Meiotic DSBs are formed by the Spo11 protein, an endonuclease that is homologous to type II topoisomerases [3]. DSBs are formed by a mechanism involving the covalent linkage of Spo11 attached to the 5′-ends of DSBs. These Spo11 proteins are removed by endonuleolytic cleavage involving the Mre11/Rad50/Xrs2 complex and Sae2/Com1 [4].

The 5′ ends receive further resection, leaving 3′-ended single-strand (ss) DNA tails [5]. These ssDNA are the substrate for Rad51 and Dmc1, eukaryotic RecA homologs, which catalyze the homology searching and strand exchange reactions [2]. Rad51 is essential for both mitotic and meiotic recombination whereas Dmc1 is a meiosis-specific protein; both RecA homologs are essential for repairing meiotic DSBs. Unrepaired DSBs with extensive 3′-ended ssDNA accumulate in the absence of Dmc1 and Rad51 [6].

The location and timing of DSB formation are highly controlled during meiosis [7], [8]. DSBs are preferentially formed at regions that are free of nucleosome structures, typically found in the areas upstream of transcription start sites [9]. This location specificity can be explained by the accessibility of chromatin to DSB-forming enzymes. The timing of DSB formation is controlled under multilayers of mechanisms, including transcription, splicing and post-translational modifications [9]. The *MER2* gene encodes one of the essential ancillary factors of Spo11 and is spliced in a meiosis-specific manner. The resultant protein is regulated through phosphorylation by Cdc28 and DDK, two major kinases that are essential for cell cycle control [10]–[12].

Certain non-null alleles of the *RAD50* and *MRE11* genes and deletion of the *SAE2/COM1* gene results in the accumulation of DSBs with Spo11 covalently attached to their 5′ ends; this covalent linkage prevents the processing of DSB ends [13]. These mutants are collectively referred to as “*rad50S*-like” hereafter. In contrast, when two RecA homologs, Rad51 and Dmc1, are defective, DSB ends receive extensive processing, leaving long 3′-tailed ssDNA [14]. In the *rad50S*-like mutants, much fewer DSBs are formed than in *dmc1* [6], [15], [16]. Such mutants show uneven DSB distribution along chromosomes, creating domains with very few or no DSBs (i.e., cold spots). On the other hand, in *dmc1* and *rad51 dmc1* mutants, the situation is similar to wild type in that DSBs are more evenly distributed throughout chromosomes [6], [16].

The Spo11 protein tagged with a combination of hemagglutinine epitope and a hexahistidine sequence (hereafter called *spo11-HA*) is not fully functional, leading to a reduction in DSB formation. Typically, the number of DSBs in such mutants is up to 80% of wild type [17]. In this and other hypomorphic *spo11* mutants showing various levels of reduction in DSB formation, the level of crossovers tends to be maintained at the expense of noncrossovers. This phenomenon is called crossover homeostasis [17].

The *PCH2* gene (Pachytene CHeckpoint), which encodes a putative AAA+ ATPase, is important in evoking the meiotic cell cycle arrest/delay when homologous recombination intermediates accumulate [18]–[21]. Pch2 also plays a role in the morphogenesis of meiotic chromosome axes [22], and chromatid-partner choice in meiotic DSB repair [21]–[23]. In addition, Pch2 is required for maintaining the integrity of rDNA repeats during meiosis by suppressing homologous recombination [24]. Pch2 interacts with Xrs2 and functions in the Tel1 pathway of the recombination checkpoint [23]. Furthermore, extensive genetic analyses showed that crossing over is elevated in medium and large chromosomes in the *pch2* mutant [25]. Crossover interference is compromised and the ratio of crossovers to noncrossovers is elevated [25], [26]. Taken together, it is proposed that Pch2 is involved in making a decision of forming crossovers versus non-crossovers and in imposing crossover interference [25].

Here we discovered an unexpected link between meiotic DSB formation and Pch2 function. In the *sae2* mutant background, the absence of Pch2 leads to a substantial reduction in DSB formation. This trend is more pronounced in the bigger chromosomes than the smaller chromosomes. Interestingly, the absence of Pch2 only mildly affects DSB formation when the *dmc1* or *rad51 dmc1* mutant background was employed. Consistent with its involvement in DSB formation, the number of DSBs was further reduced when the *pch2* mutation was combined with *spo11-HA*, a hypomorphic allele of *SPO11*. Taken together, we propose a novel function of Pch2 at the stage of initiating meiotic recombination.

## Results

### The *pch2* mutation causes a reduction in DSB formation

**Figure 1:**
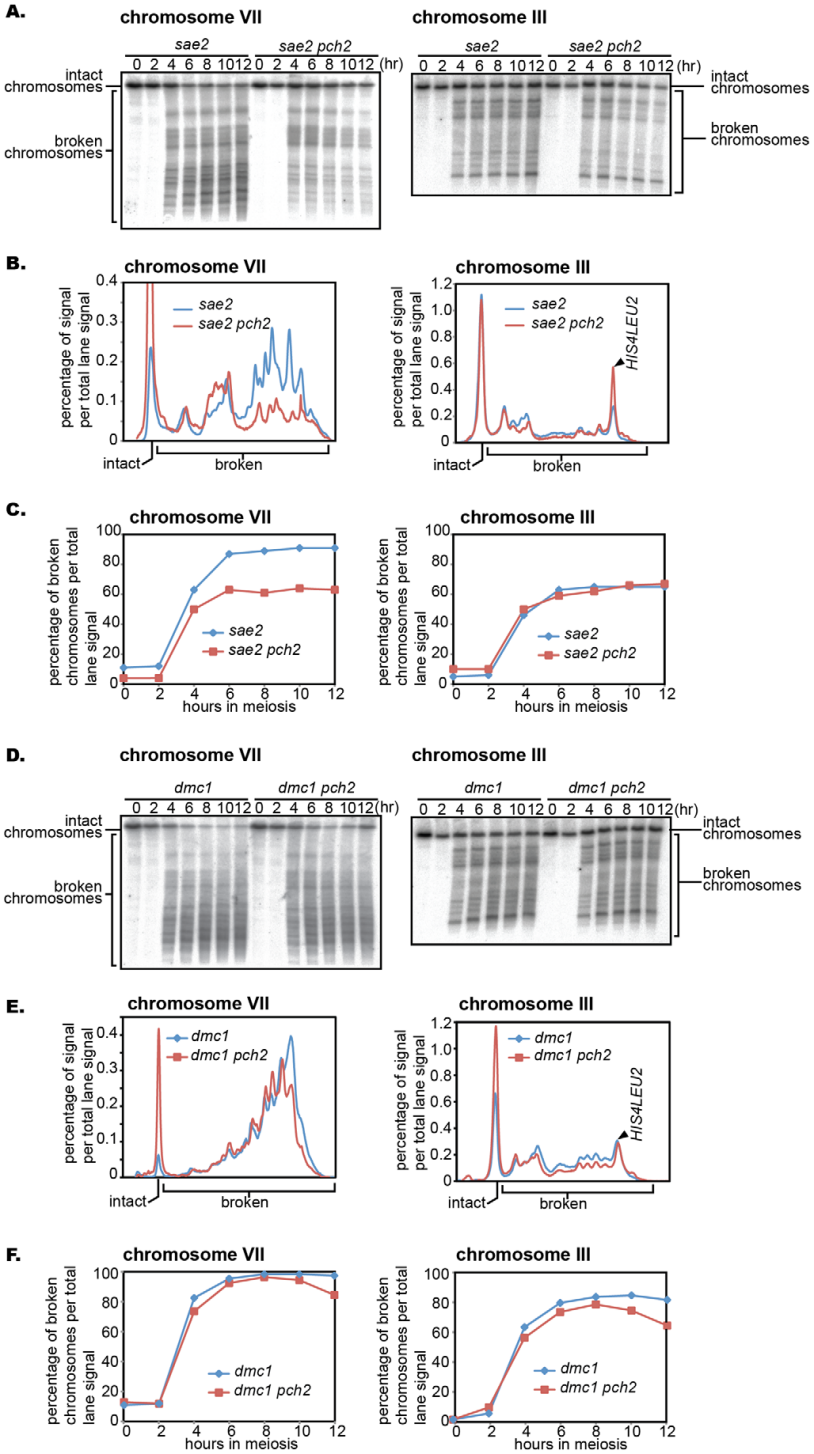
DSB formation is reduced by the *pch2* mutation and the effect is chromosome specific. (A, D) Diploid *sae2* and *sae2 pch2* mutants (A) and *dmc1* and *dmc1 pch2* mutants (B) were introduced into meiosis and DSB formation was detected at indicated time points in chromosomes VII and III (see Materials and Methods for details). (B, E) Lane profiles of chromosome VII and III when amount of broken chromosomes are maximum. Lane profiles of 10 and 12 hours in each mutant background were normalized and averaged to obtain the profiles shown (see Materials and Methods for details). (C, F) DSB formation kinetics along meiotic time course. DSB formation was expressed as the fraction of the sum of signals corresponding to broken chromosomes per total lane signal. Cells from the same time course were used to examine both chromosomes VII and III.

The *pch2* mutation was originally isolated as a suppressor mutation that bypasses the cell cycle arrest caused by the *zip1* mutation. The *pch2* mutation bypasses a group of mutations in which recombination intermediates accumulate during meiosis. In such mutants, the *spo11* mutation usually suppresses the cell cycle arrest phenotype because it gets rid of meiotic recombination itself, thus leading to no accumulation of unrepaired DSBs. We considered the possibility that the *pch2* mutation might bypass the cell cycle arrest of various recombination mutants by reducing meiotic DSB formation, the initiator of meiotic recombination. We took advantage of the *sae2* mutant, one of the *rad50S*-like mutants in which meiotic DSBs accumulate with the Spo11 protein covalently bound to DSB ends. The efficiency of DSB formation in a given chromosome was evaluated by using pulsed-field gel electrophoresis (PFGE), followed by Southern blotting with probes specifically recognizing the ends of specific chromosomes. The *sae2* single mutant and the *sae2 pch2* double mutant diploids were used. Throughout the time course of the *sae2 pch2* double mutant, a substantial reduction in DSB formation was detected in chromosome VII (Figure 1 A to C, left). Both strains showed similar kinetics regarding DSB formation, with a plateau being reached approximately 6 hours after the induction of meiosis. This result was unexpected because a previous report showed no sign of DSB reduction in the *pch2* mutant [20]. In that study, an artificial *HIS4LEU2* hotspot in chromosome III was used as a model system. This prompted us to examine DSBs in chromosome III. Consistent with previous reports, a very similar level of DSB formation was detected in both mutants (Figure 1 A to C, right). We did notice a slight difference in the profile of broken chromosomes, with a trend that the level of DSBs at the *HIS4LEU2* hotspot was more pronounced in the *sae2 pch2* double mutant. The *pch2* mutation suppresses the cell cycle delay phenotype caused by the *rad50S*-like mutation [20], and it is possible that the reduction in DSB formation found in the *pch2* mutant is associated with the accelerated cell cycle progression. To test this possibility, DSB formation was examined in the *ndt80* background, in which the meiotic cell cycle does not exit from the pachytene stage of prophase I (Figure S1). A similar reduction in DSB formation was seen in the *pch2* mutation in the *ndt80* background as well, arguing that this phenotype is more closely associated with the *pch2* mutation itself.

**Figure 2:**
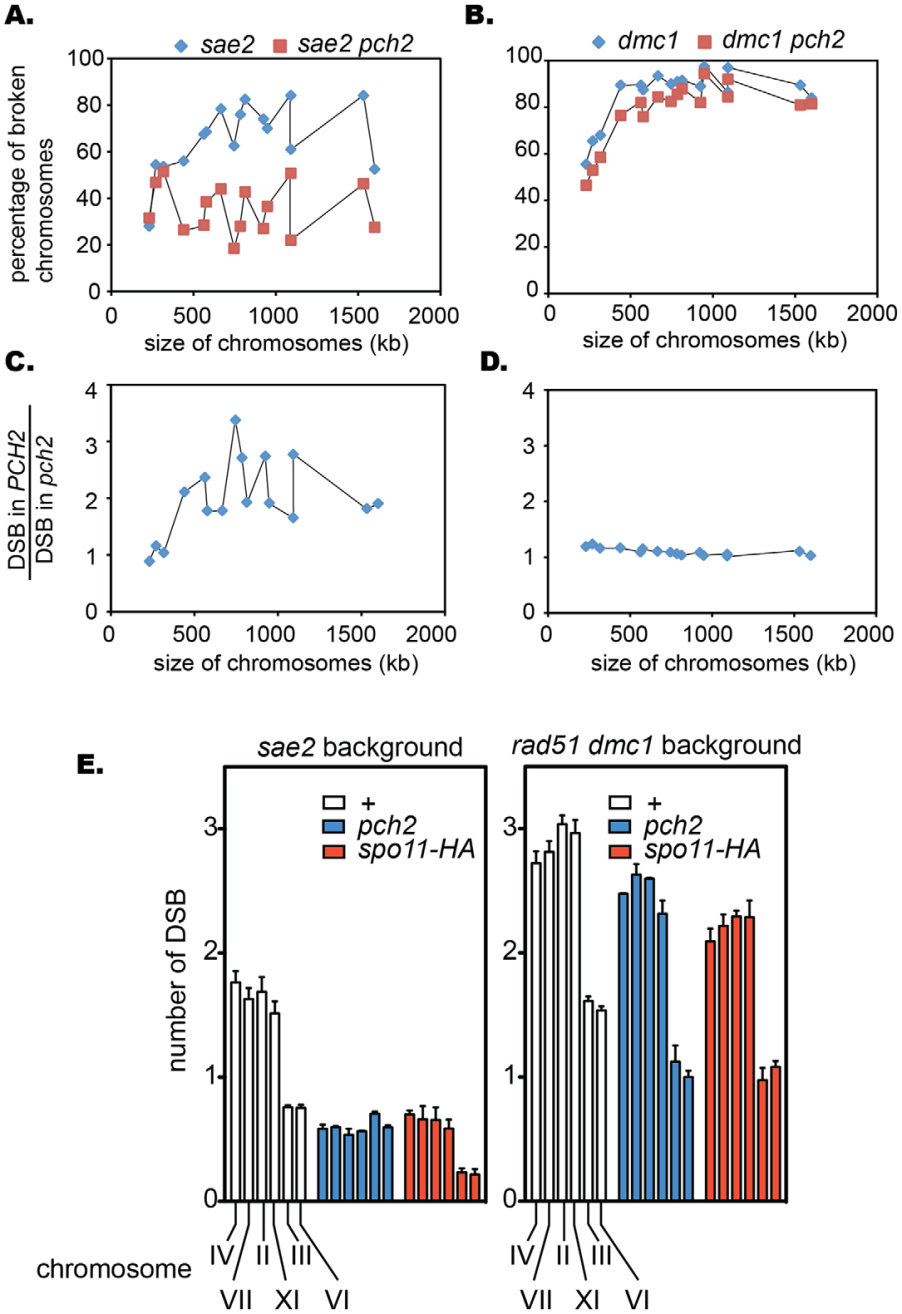
Quantitative analysis of the effect of the *pch2* mutation on DSB formation. (A, B) the relationship between DSB formation and chromosome size in the *sae2* (A) and *dmc1* (B) backgrounds. DSB formation was expressed by the percentage of broken chromosomes (see Figure 1C and F, and Materials and Methods). Welch two sample t-test was used to analyze the effect of the *pch2* mutation on DSB formation. The break rate on each chromosome was deduced from the procedure described in Materials and Methods and Supplemental material. The effect of the *pch2* mutation on DSB formation is significant in both the *sae2* background (t = 3.8661, p-value  = 0.0005509) and the *dmc1* background (t = 2.5528, p-value  = 0.01707). (B, D) DSB amount in the *PCH2* positive strain was divided by that of the *pch2* negative counterpart per each chromosome in either *sae2* (C) or *dmc1* (D). (E) Quantitative evaluation of the *pch2* effect on DSB formation. The number of DSB forming events was calculated as described in Materials and Methods and Supplemental material. At least two experiments were done for each data point. Error bars represent standard error.

The difference in the reduction of DSB formation between *sae2* and *sae2 pch2* for chromosomes VII and III raised the possibility that the *pch2* effect on DSB formation might vary between different chromosomes. Thus we decided to examine DSB formation in all the chromosomes. Overall, a substantial reduction in DSB formation, ∼ 2- to 3-fold (based on the ratio between intact versus broken chromosomes), was caused by the *pch2* mutation throughout the chromosomes, with a few exceptions; the *pch2* mutation had a subtle effect on small chromosomes I, VI, and III (Figure 2A, C). The *pch2* effect on DSB formation was quantitatively examined further in chromosomes IV, VII, II, XI, III and VI (Figure 2E, also see Materials and Methods). DSB formation was reduced by ∼ 60% to 70% in chromosomes IV, VII, II, and XI, whereas chromosomes III and VI showed a 10% and 20% reduction respectively.

### The *pch2* effect on DSB formation is largely alleviated in the *dmc1* or *rad51 dmc1* mutant background

In the *dmc1* mutant, the amount and distribution of DSBs are closer to those of wild type than in *sae2* [6]. Thus, we decided to examine the effect of the *pch2* mutation on DSB formation in the *dmc1* mutant background. In the *dmc1* mutant background, DSBs are more efficiently formed than the *sae2* mutant background, consistent with previous reports [6], [15], [16]. Moreover, unlike in the *sae2* mutant background, the *pch2* mutation effect was largely alleviated. The amount of DSBs and the timing of DSB formation were similar between the *dmc1* single and the *dmc1 pch2* double mutants, although the *dmc1 pch2* mutant consistently showed a mild reduction in DSB formation (Figure 1 D to F). This is more clearly demonstrated by the comparison of lane profiles of Southern blots (Figure 1E), where the position of the highest peak of the fragmented DNA in *dmc1* shifts towards the left in *dmc1 pch2*, corresponding to larger fragments of DNA. In the *dmc1* mutant background, we did not see any correlation between the reduction of DSB formation and chromosome size: the ratio of broken chromosomes between the *dmc1* single and the *dmc1 pch2* double mutants is almost constant throughout all the chromosomes (Figure 2B, D).

**Figure 3:**
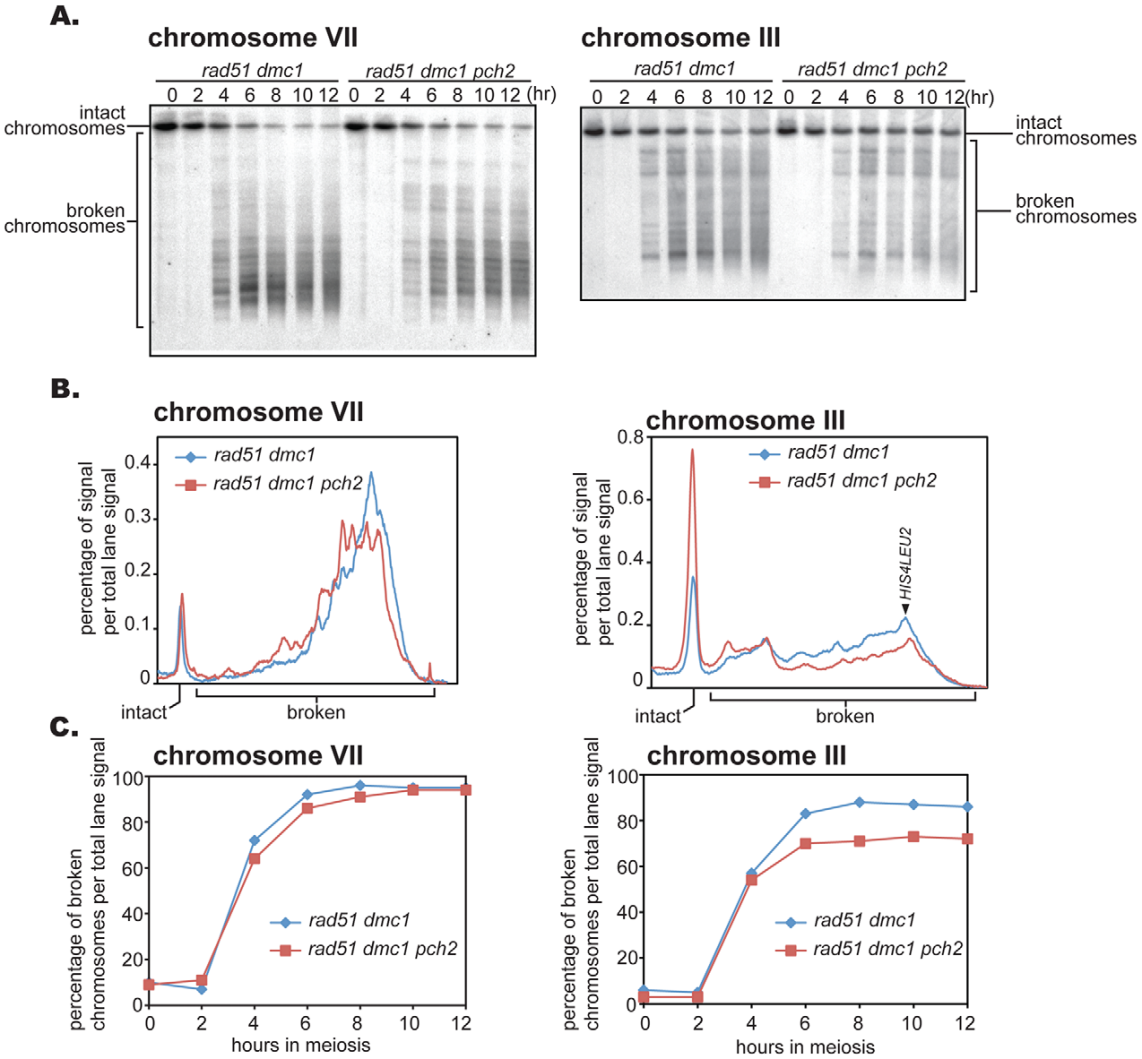
The *pch2* mutation mildly reduces DSB formation in the *rad51 dmc1* mutant background. (A) Diploid *rad51 dmc1* and *pch2 rad51 dmc1* mutants were introduced into meiosis and DSB formation was detected at indicated time points in chromosomes VII and III. (B) Lane profiles of chromosome VII and III when amount of broken chromosomes are maximum. Lane profiles of 10 and 12 hours in each mutant background were normalized and averaged to obtain the profiles shown. (C) DSB formation kinetics along meiotic time course. DSB formation was expressed as the fraction of the sum of signals corresponding to broken chromosomes per total lane signal. Cells from the same time course were used to examine both chromosomes VII and III.

It was recently reported that *PCH2* is involved in inhibiting homologous recombination between sister chromatids [21], [23]. This raises the possibility that the amount of broken chromosomes we detected in *pch2 dmc1* could be an underestimate of the total number of breaks. Thus, we also employed the *rad51 dmc1* double mutant background in which genes encoding two major RecA homologs are deleted; very few DSBs, if any, are repaired, even when the mechanism that normally inhibits the Rad51-dependent pathway is compromised. Similar to the *dmc1* background, *pch2* only moderately reduced DSB formation in the *rad51 dmc1* mutant background in chromosome VII, and the difference is slightly more pronounced in chromosome III (Figure 3). Along with chromosomes VII and III, four more chromosomes, IV, II, XI and VI, were quantitatively examined (Figure 2E). There was an reduction of 20% on average in DSB formation.

### DSB formation is further reduced by combining *pch2* and *spo11-HA*

**Figure 4:**
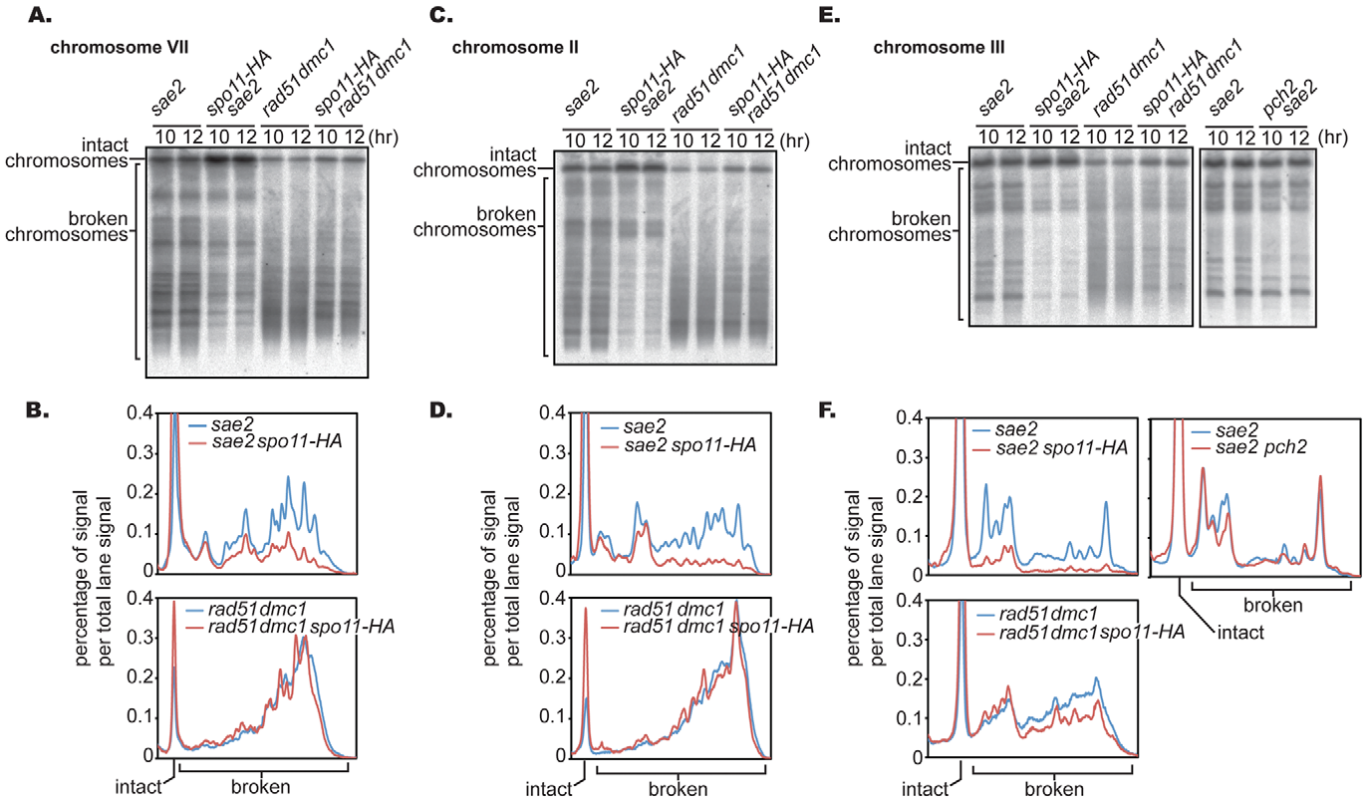
*spo11-HA* effect on DSB formation is more pronounced in *sae2* than in *rad51 dmc1*. (A, C, E) Southern blot images of accumulated broken chromosomes. (B, D, F) Lane profiles of the Southern blots above. Lane profiles of 10 and 12 hours in each mutant background were normalized and averaged to obtain the profiles shown. At least two experiments are done for each genotype and a representative result is shown here. Cells from the same time course were used to examine these chromosomes and those in Figure S3. See Figure 2E for quantitative analysis of DSB formation.

Spo11-HA is not fully functional and caused a reduction in meiotic DSB formation, which was evaluated physically in the *rad50S* background or genetically by measuring intragenic recombination between heteroalleles in return-to-growth assays [17]. We asked if the reduction of DSB formation caused by the *spo11-HA* allele shows a similar phenotype to *pch2*. The *spo11-HA* allele was combined with either the *sae2* mutation or the *rad51 dmc1* double mutant, and meiotic DSB formation was examined. The effect of *spo11-HA* was compared quantitatively with that of *pch2* (Figure 2E). Consistent with previous studies, DSB formation was reduced in the *sae2* background in all six chromosomes examined (VII, II, III, IX, XI and VI) (Figures 4, S2 and 2E). The overall average reduction was 74%. Unlike *pch2*, reduction was observed in small chromosomes III and VI as well (Figure 4 EF, S2 and 2E). In contrast, in the *rad51 dmc1* double mutant background, the effect of *spo11-HA* was substantially alleviated (27% reduction on average); this trend is similar to what was seen in the *pch2* mutant (Figure 4, S3 and 2E)

**Figure 5:**
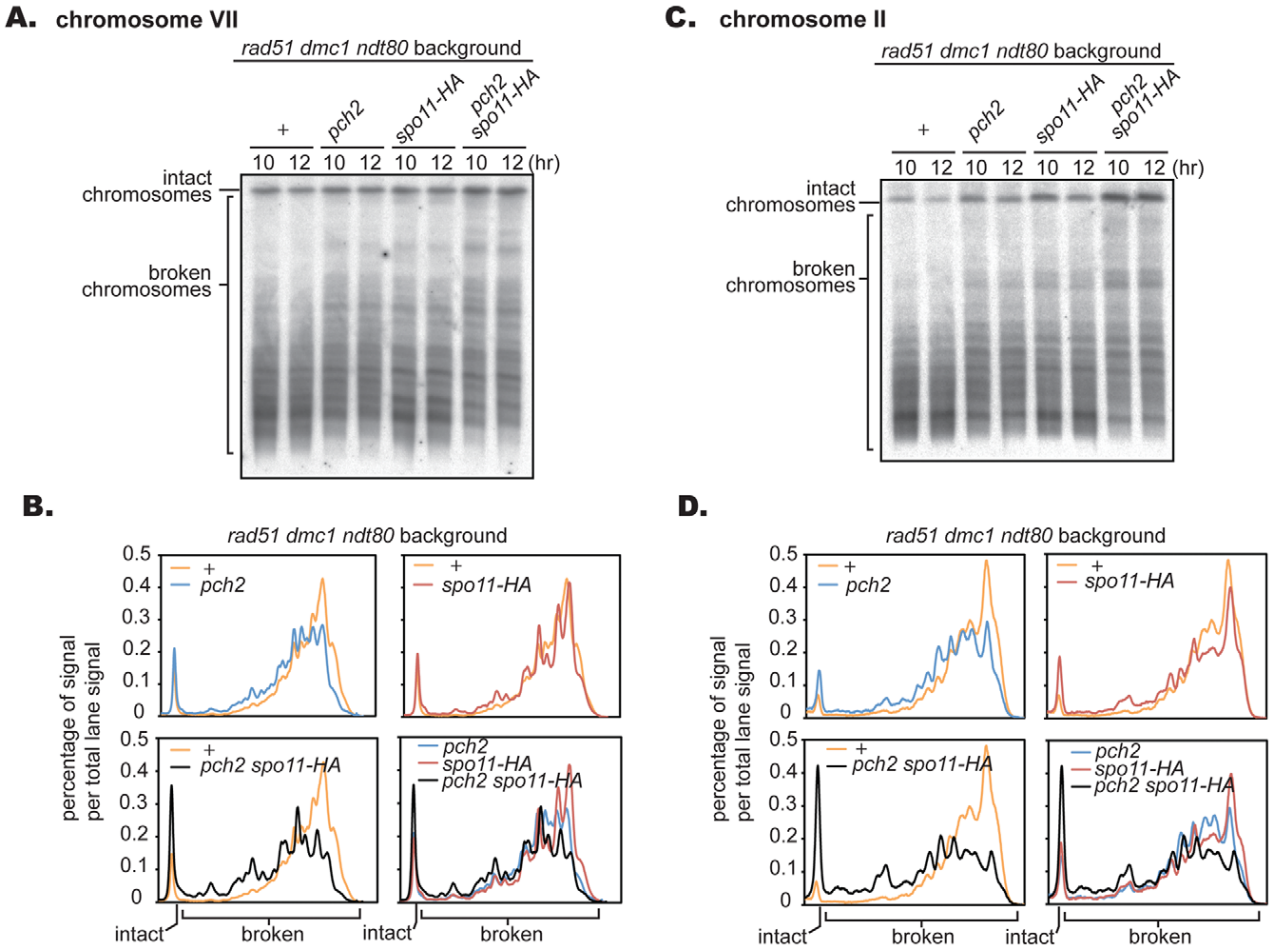
The combination of *pch2* and *spo11-HA* further reduces DSB formation. (A) Diploid *rad51 dmc1*, *pch2 rad51 dmc1, spo11-HA rad51 dmc1* and *pch2 spo11-HA rad51 dmc1* mutants were introduced into meiosis and DSB formation was detected at indicated time points in chromosomes VII and II. To avoid any effect caused by unscheduled cell cycle progression, experiments were done using the *ndt80* mutant background (see text for further details). (B) Lane profiles of chromosome VII and II when amount of broken chromosomes are maximum. Lane profiles of 10 and 12 hours in each mutant background were normalized and averaged to obtain the profiles shown. At least two experiments were done for each genotype and a representative result is shown here. Cells from the same time course were used to examine both chromosomes.

To examine the relationship between DSB reduction caused by the *pch2* and *spo11-HA* mutations, the two mutations were combined and the impact on DSB formation was tested in the *rad51 dmc1* mutant background. The *ndt80* mutation was employed to rule out the possibility that the reduction in DSB formation is caused indirectly by unscheduled cell cycle progression. The reduction in DSB formation was further pronounced when these two alleles were combined, in both chromosome VII and II (Figure 5), supporting the idea for the involvement of Pch2 in DSB formation and providing evidence for the argument that the reduction in DSB formation by *spo11-HA* and *pch2* is induced through different mechanisms.

## Discussion

In this report, we present evidence that Pch2 is involved in meiotic DSB formation.

There are two interesting features regarding the *pch2* phenotypes. First, DSB formation reduction is most prominent in the *sae2* background, while the effect becomes much milder in the *rad51 dmc1* double mutant background. Second, the degree of reduction in DSB formation varies between chromosomes, with minimum effect in small chromosomes I, VI and III.

### Pch2 and the bypass of cell cycle arrest

The characterization of genes involved in DNA damage surveillance mechanisms during meiosis (i.e., the recombination checkpoint, also known as the pachytene checkpoint) has been done using mutants defective in steps after meiotic DSBs are formed, including *rad50S*-like mutants, *zip1* mutants, and *dmc1* mutants. The *pch2* mutant itself was isolated as a mutant that bypasses the meiotic cell cycle arrest caused by *zip1*.

The main issue in using such mutant backgrounds for studying the meiotic checkpoint is that the bypass effect can be brought about through two independent mechanisms. The amount of checkpoint signaling is obviously reduced when the mechanism to sense/transmit the signal is compromised. On the other hand, by reducing the number of DSBs formed, the same phenomenon can be observed since there will be fewer sites of DNA damage to activate checkpoint signaling. Indeed, the cell cycle arrest caused by a repair defect after DSB formation is known to be bypassed when DSB formation itself is abolished (e.g., by the *spo11* mutation).

We propose that the bypass effect of the *pch2* mutation on cell cycle arrest is caused, at least in part, by the overall reduction of DSB formation. This view is consistent with the fact that the *pch2* bypass is most prominent in certain mutants in which the checkpoint activation is relatively mild, namely *rad50S*-like mutants and “*zip*” mutants (*zip1*, *zip2* and *zip3*). The bypass is seen to a lesser extent in the *dmc1* mutant where the checkpoint pathway is activated strongly [19], [21], [27]. In *rad50S*-like mutants, the meiotic cell cycle does not arrest permanently but only slows down. This is because DSB ends are not processed, thus the Mec1-dependent pathway is not activated [28]. In *zip* mutants, the majority of DSBs are repaired [29]. It has previously been shown that meiotic cells respond to DSBs less sensitively than vegetative cells [30]. In vegetative cells, one unrepaired DSB is enough to completely arrest the cell cycle, whereas in meiosis, a DSB can slow down the cell cycle but cells can still undergo two meiotic divisions and eventually form spores with unrepaired DSBs. Taken together, the degree of meiotic cell cycle retardation is highly related to the extent of checkpoint activation, and one of the critical parameters to contribute to this activation is the amount of DSBs formed, which is attributable to the involvement of Pch2.

It was recently reported that Pch2 is involved in suppressing recombination between sister chromatids [21], [23]. The *pch2* mutation allows the *dmc1* mutant to progress through the cell cycle more efficiently, via a Rad54 dependent mechanism. Given that Pch2 is involved in DSB formation, it is possible that this phenomenon is caused by an overall reduction in DSB formation. The inhibition of intersister recombination requires activation of the pachytene checkpoint pathway that involves phosphorylation of Mek1 [31]. Rad54 is one of the target proteins of Mek1 [32]. The phosphorylation of Rad54 by Mek1 leads to the reduced interaction between Rad51 and Rad54, playing a critical part in the inhibition of intersister recombination. Thus, reduced DSB formation should lead to the overall compromise of the inhibition of the intersister recombination.

Spore inviability is known to be exacerbated when the *spo11-HA* and *pch2* alleles are combined. In this study, we showed that DSB formation is specifically compromised when the *spo11-HA* and *pch2* mutations are combined. These findings can be linked in two possible ways. First, the total number of DSBs is reduced, potentially leading to fewer crossover products. Second, decreased DSB formation leads to less inhibition of intersister recombination. Both effects can directly contribute to spore inviability.

Our proposal does not preclude the possibility of the role of Pch2 after DSB formation. Indeed, it has been proposed recently that Pch2 acts together with Xrs2 and Tel1 in meiotic checkpoint function [23], although many of the phenotypes examined, including cell cycle arrest, homologous recombination partner-choice and phosphorylation of Hop1 and Mek1, can also be explained by the overall reduction of DSBs formed. In the mouse, Trip13, the ortholog of Pch2, is necessary for completing meiotic recombination [33], [34]. In Drosophila and *C.elegans*, Pch2 is implicated in monitoring DSB-independent chromosomal defects in axes structure and chromosome synapsis respectively [35]–[37]. In Drosophila, Pch2 is localised to the outside of the nuclear membrane [36]. These results argue that Pch2 may have additional function(s) in higher eukaryotes.

### Pch2 and the chromosome size effect

DSB formation in the *pch2* mutant has been examined previously and a reduction in DSB formation has not yet been reported [19], [20], [24]. What is common in these previous experiments is either that the DSB formation was monitored only at the *HIS4LEU2* hotspot or that the *dmc1* mutant background was used (or both). The *HIS4LEU2* hotspot is on chromosome III, and, consistent with the previous data, we did not find any reduction in DSB formation at this locus either (on the contrary, we found that DSB formation was slightly elevated at this locus). In the *dmc1* mutant background, the *pch2* effect on DSB formation is very mild. Thus, our observations are consistent with what has previously been reported by other groups.

We propose that Pch2 is important for promoting DSB formation, especially in the larger chromosomes. In the absence of Pch2, a similar level of DSB formation, 0.6 per chromosome on average, is found throughout six chromosomes closely examined in the *sae2* background (Figure 2E). This is distinct from the case of *spo11-HA* in which DSB formation is rather uniformly reduced regardless of the size of chromosomes. This might indicate that bigger chromosomes need an additional Pch2-dependent mechanism to further facilitate DSB formation. What could this be? One possibility is that such Pch2 functionality is related to DNA replication. Once a replication origin is fired, DNA needs to be replicated for DSBs to be formed [38], [39]. Local delay in DNA replication causes delayed DSB formation. Formation of such late DSBs is specifically reduced when the *rad50S-like* mutant background is employed [6], [38]. Furthermore, Pch2 interacts with Orc1, a component of the origin recognition complex [24]. Thus, it is possible that, in bigger chromosomes, there are regions where efficient replication or origin firing requires Pch2, with the number of such regions roughly related to the size of chromosomes.

A recent publication has implicated the role of Pch2 in suppressing DSB formation at the border of the repetitive ribosomal DNA arrays [24]. Together with our observation, it is likely that Pch2 functions in both inducing and suppressing DSB formation. We found similar levels of DSB formation in small chromosomes in both *pch2* and *pch2 sae2* mutants. In light of both positive and negative roles for Pch2 in DSB formation, it is possible that, in the *pch2* mutant, DSB formation at certain locations is derepressed while DSB formation in general becomes less efficient, leading to a similar level of DSB formation to that of the *PCH2* positive background. Overall, however, we found very similar distributions of DSBs along the length of small chromosomes in *sae2* and *sae2 pch2* mutants (Figure 1AB, Figure S3 chromosomes III and VI), arguing that the involvement of Pch2 in facilitating DSB formation is minor in small chromosomes. However, we did notice that DSB formation at the *HIS4LEU2* hotspot is enhanced specifically in *sae2 pch2*, arguing for the DSB-suppressive role of Pch2 at this locus (Figure 1AB). Whether or not such a DSB-suppressive role of Pch2 at *HIS4LEU2* is carried out through the same mechanism as in the ribosomal DNA locus remains to be uncovered.

### Pch2 and the control of crossing over

The amount and distribution of meiotic DSBs differs, depending on what recombination mutant backgrounds are employed [6], [15], [16]. In the *rad50S*-like mutant, overall DSB levels are lower than in the *dmc1* or *rad51 dmc1* mutant, especially with much broader “cold” regions proximal to centromeres and telomeres. Thus, we propose to categorise DSBs into two classes: those visible in the *rad50S*-like mutant, or preprocessing DSBs (since DSBs are not processed in the *rad50S*-like mutant); and DSBs formed after the preprocessing DSBs are processed, or postprocessing DSBs. In this scenario, the total amount of DSBs is the sum of preprocessing and postprocessing DSBs, and can be measured by using the *rad51 dmc1* mutant.

We showed that the number of preprocessing DSBs was substantially reduced while the total amount of DSBs formed was only mildly affected in the absence of Pch2. How can Pch2 impact specifically on preprocessing DSB formation? One possibility is that DSB formation is modulated by a Pch2-dependent feedback control, which is possibly associated with the Tel1-dependent pathway. A close association between Pch2 and Tel1/Mre11-Rad50-Xrs2 has recently been suggested [23]. If they form a positive feedback that facilitates DSB formation, the absence of such feedback should lead to a reduction in DSB formation. Since DSB processing causes a more robust response using Mec1, this can create a stronger positive feedback to facilitate DSB formation, making the reduction in DSB formation caused by the *pch2* mutation less conspicuous.

Pch2 is proposed to play a role in the control of crossover formation and distribution [25]. Intriguingly, in *pch2*, genetic analyses showed that elevated levels of crossovers are formed in bigger chromosomes (chromosomes VII, VIII and VX) with little difference in a small chromosome (chromosome III) [25], [40] (we did notice that there is a discrepancy between [25], [40] and [26] regarding this point). Based on our scenario with two kinds of DSB formation, the ratio between preprocessing DSBs and postprocessing DSBs is changed in *pch2* while the total number of DSB formation is similar to wild type. The ratio should vary in a size dependent manner among chromosomes, with similar ratios to those in wild type in small chromosomes, and a higher fraction of postprocessing DSBs in bigger chromosomes. Along with our results, we propose that preprocessing DSBs and postprocessing DSBs follow distinct destinies regarding the formation of crossovers. We propose that postprocessing DSBs are more likely to be repaired to form crossovers than preprocessing DSBs. In this scenario, in *pch2*, crossover formation in small chromosomes is unchanged because the ratio and amount of pre- and postprocessing DSBs are maintained, while large chromosomes have more postprocessing DSBs than in wild type, leading to the production of more crossovers. This could be related to the activation of the meiotic recombination checkpoint. This activation is essential for building up the bias for interhomolog recombination. Preprocessing DSBs might have a role in igniting the checkpoint pathway so that subsequently formed postprocessing DSBs are more efficiently converted into interhomolog crossovers.

In the *spo11-HA* mutant, preprocessing DSB formation is reduced, whereas postprocessing DSB formation is less affected, similar to the situation in *pch2*. *spo11-HA* was one of the alleles used to propose crossover homeostasis, the mechanism to maintain the amount of crossovers even when the number of meiotic DSBs is limited [17]. Given that meiotic DSBs detected in the *rad51 dmc1* double mutant are a good reflection of DSB formation in wild type [6], [16], the reason why the *spo11-HA* mutant produces efficient crossovers may be because a reasonable amount of DSBs are eventually formed. Furthermore, based on previously published literature, the *spo11-HA* mutant tends to show higher levels of crossovers than wild type (e.g., Figure 1C in [17] and Figure 2B in [25]), with a higher crossover/noncrossover ratio (Figure 2 in [17]). These alterations could also be due to the changed ratio between pre- and postprocessing DSBs in *spo11-HA*, although the level of change is not as prominent as in *pch2*.

## Materials and Methods

### Yeast strains

Genotypes of yeast strains are given in Table S1. All yeast strains used are isogenic derivatives of SK1. All markers were introduced by transformation and by genetic crosses between transformants and/or existing strains. The ORFs of *RAD51*, *DMC1*, *PCH2*, *SAE2* were replaced with drug resistant markers by PCR mediated gene disruption [41]. *rad51::URA3*, *spo11-HA*, *ndt80::LEU2* were previously described [17], [42], [43].

Strains used are: TBR5514, 5515, 5461 and 5462 in Figure 1; TBR5514, 5515, 5461, 5462, 5520, 4664, 5952 and 5954 in Figure 2; TBR5520 and 4664 in Figure 3; TBR5514, 5952, 5520, 5954 and 5515in Figure 4; TBR6192, 6194, 6396 and 6397 in Figure 5; TBR6618 and 6619 in Figure S1; TBR5514, 5515, 5952 in Figure S2; TBR5514, 5952, 5520 and 5954 in Figure S3.

### Meiotic time course and detection of meiotic DSBs

SK1 strains were introduced into meiosis as described previously with minor modifications [44]. Briefly, cells from a saturated culture in YPD supplemented with adenine (0.3 mM) and uracil (0.2 mM) were diluted in buffered YTA media (1% yeast extract, 2% tryptone, 1% potassium acetate, 50mM potassium phthalate) [24], and incubated for 12 hours. The pre-sporulation culture was washed once with water, and resuspended in 2% potassium acetate. Cells were harvested at appropriate time points and stored at −80°C until use.

Meiotic DSBs were detected as described previously with minor modifications [45]. Briefly, genomic DNA was prepared inside agarose plugs and separated on PFGE (120°, 14°C, 24 hours at 6V/cm). Switching times applied are: 5 to 30 seconds for chromosome I, VI and III, and 20 to 60 seconds for the rest. Separated DNA was subjected to Southern blotting, with each chromosome visualized using radio-labeled probes annealing specifically to the chromosome (see below). The radio-labeled membrane was imaged by a phosphoimager (Fuji, FLA5100). The obtained images were background-subtracted using AIDA (Raytest), an image analysis software, and the lane profiles were exported and further analysed using Excel (Microsoft). Normalized lane profiles were obtained with each point divided by the total amount of signal per lane. Either 8 and 12 hour or 10 and 12 hour lane profiles were averaged to obtain the lane profiles presented. DSB formation reaches a plateau around 6 hours, thus the differences between 8, 10 and 12 hour lane profiles are minimal (Figure 1). In Figure 2A to D, data points for chromosomes IV, VII, II, XI, III and VI are the average of four independent experiments in *sae2* and two in *sae2 pch2*. Other data points were taken once.

Probes for Southern blotting were prepared by the random priming method with the following region of DNA as templates; chromosome I, 14109–14868; II, 12814–13476; III, 12020–12746; IV, 1519209–1519937; V, 564921–565748; VI, 257224–257974; VII, 14960–16249; VII, 12926–13616; XI, 427880–428685; X, 733971–734806; XI, 12712–13370; XII, 16639–17613; XIII, 12681–13456; XIV, 756008–756808; XV, 325259–325946; XVI, 925187–926861. All probes anneal to an end of a given chromosome except the one for chromosome XV, which anneals near the centromere.

### Evaluation of DSB formation efficiency

Calculations to obtain the estimated DSBs on chromosome used in Figure2E were done as follows.

Let N be the number of DSBs on a chromosome of the size T. Due to the constraint in the experiments, we can only observe DNA fragments sharing one end of a chromosome even when two or more breaks occur simultaneously on the chromosome. Thus, actual N is unknown and to be estimated. Let X1 be the size of DNA fragments sharing one end of a chromosome, and let h(x) be the hazard rate function, defined by
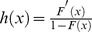
where F(x) is the probability distribution of X1 and F'(x) is its derivative. The hazard rate function is intensively studied in survival analysis and represents the instantaneous break rate when there is no other DSB between the end of the chromosome and x. With an assumption that DSBs occur independently of each other, the break rate of DSBs can be approximated by averaging the observable hazard rate function. Thus, the expected number of DSBs is obtained by


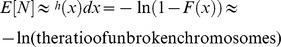


For further details of this approximation, see Text S1.

## Supporting Information

Figure S1 **The** ***pch2*** **mutation reduces DSB formation in the** ***sae2 ndt80*** **background.** *sae2 ndt80* and *sae2 pch2 ndt80* diploids were introduced into meiosis and DSB formation was detected at indicated time points in chromosomes VII and II. Lane profiles of the Southern blot for each mutant are shown on the right. At least two experiments were done for each genotype and a representative result is shown here. Cells from the same time course were used to examine both chromosomes.
(TIF)

Figure S2 **The effect of the** ***pch2*** **mutation on DSB formation is different from that of** ***spo11-HA*** **.** Experiments are done in pairs (i.e., *sae2* and *sae2 pch2*, and *sae2* and *sae2 spo11-HA*). Southern blot images of accumulated broken chromosomes along with normalized lane profiles are shown. Chromosome III data of *sae2* and *sae2 pch2* are the same as the one used in Figure 4E. *sae2* and *sae2 spo11-HA* data are the same as part of those presented in Figure 4 and S3.
(TIF)

Figure S3 ***spo11-HA*** **effect on DSB formation is more pronounced in** ***sae2*** **than in** ***rad51 dmc1.*** (A, C, E) Southern blot images of accumulated broken chromosomes. (B, D, F) Lane profiles of the Southern blots above. Lane profiles of 10 and 12 hours in each mutant background were normalized and averaged to obtain the profiles shown. At least two experiments were done for each genotype and a representative result is shown here. Cells from the same time course were used to examine these chromosomes and those in Figure 4.
(TIF)

Table S1 **Strains used in this work.**
(PDF)

Text S1 **Estimating DSB number in chromosomes.**
(PDF)
