# Correction: Budding Yeast Pch2, a Widely Conserved Meiotic Protein, Is Involved in the Initiation of Meiotic Recombination

**DOI:** 10.1371/annotation/93bcdc6c-e844-4d41-a9c1-40b1791d49cf

**Published:** 2012-10-19

**Authors:** Sarah Farmer, Eun-Jin Erica Hong, Wing-Kit Leung, Bilge Argunhan, Yaroslav Terentyev, Neil Humphryes, Hiroshi Toyoizumi, Hideo Tsubouchi

There are errors in the equations of the Materials and Methods section. Correct versions of the equations can be seen here:


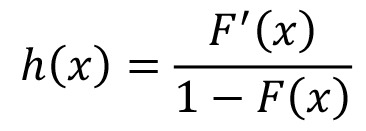


[^]


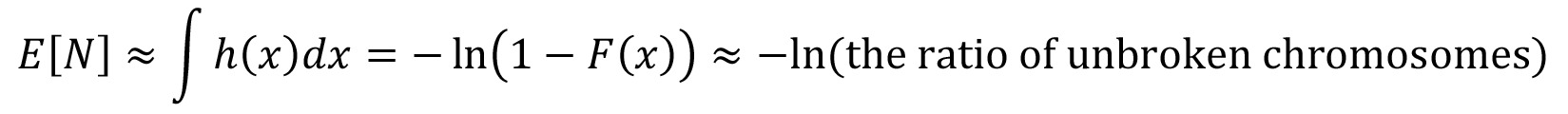


[^] 

