# Supplementary figures and images for: Budding Yeast Pch2, a Widely Conserved Meiotic Protein, Is Involved in the Initiation of Meiotic Recombination
Source: PLoS One. 2012 Jun 22;7(6):e39724. doi: 10.1371/journal.pone.0039724 (PMC3382142; doi:10.1371/journal.pone.0039724)

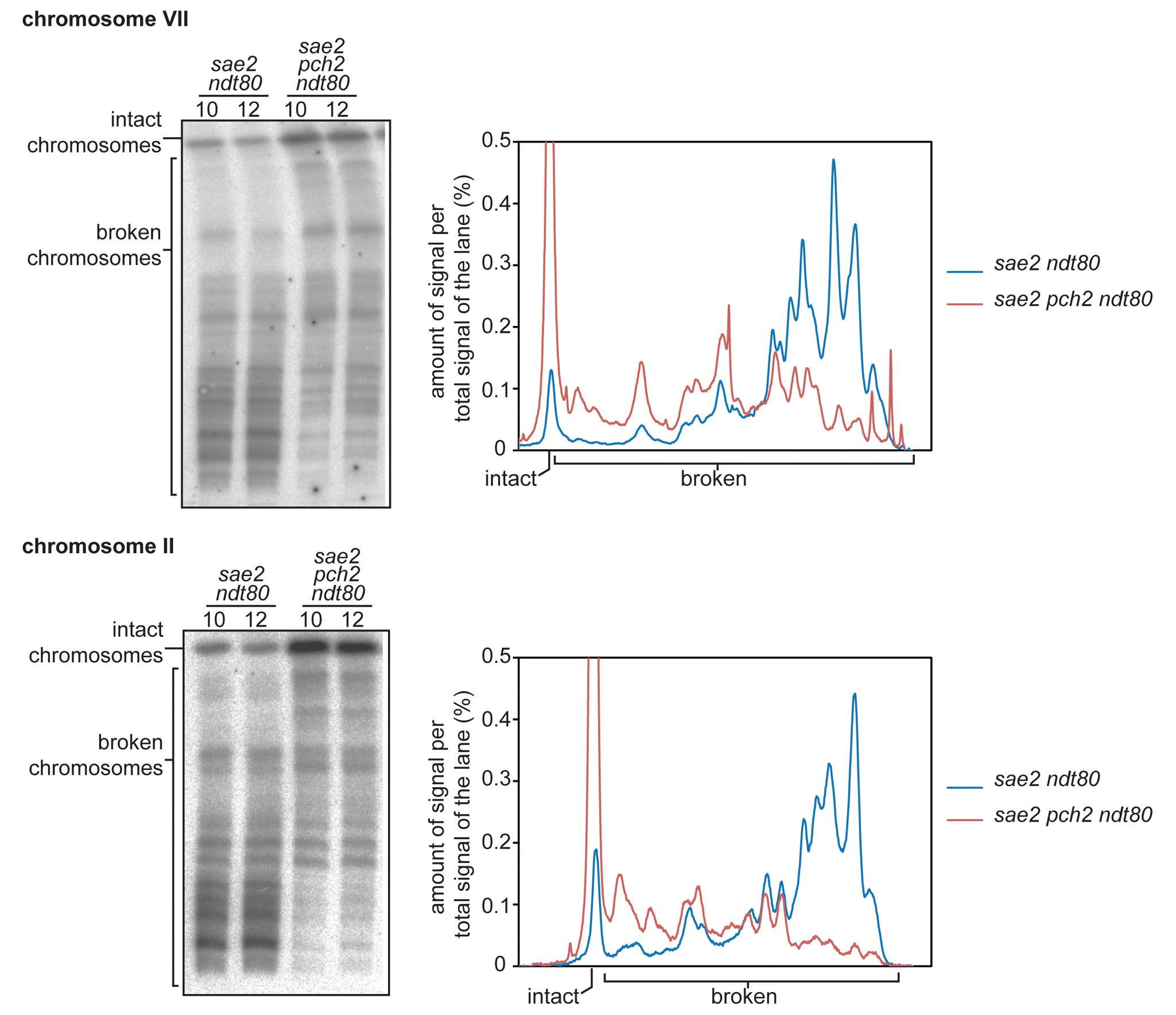

Supplement: Figure S1 — The pch2 mutation reduces DSB formation in the sae2 ndt80 background. sae2 ndt80 and sae2 pch2 ndt80 diploids were introduced into meiosis and DSB formation was detected at indicated time points in chromosomes VII and II. Lane profiles of the Southern blot for each mutant are shown on the right. At least two experiments were done for each genotype and a representative result is shown here. Cells from the same time course were used to examine both chromosomes. (TIF) [file pone.0039724.s001.tif]

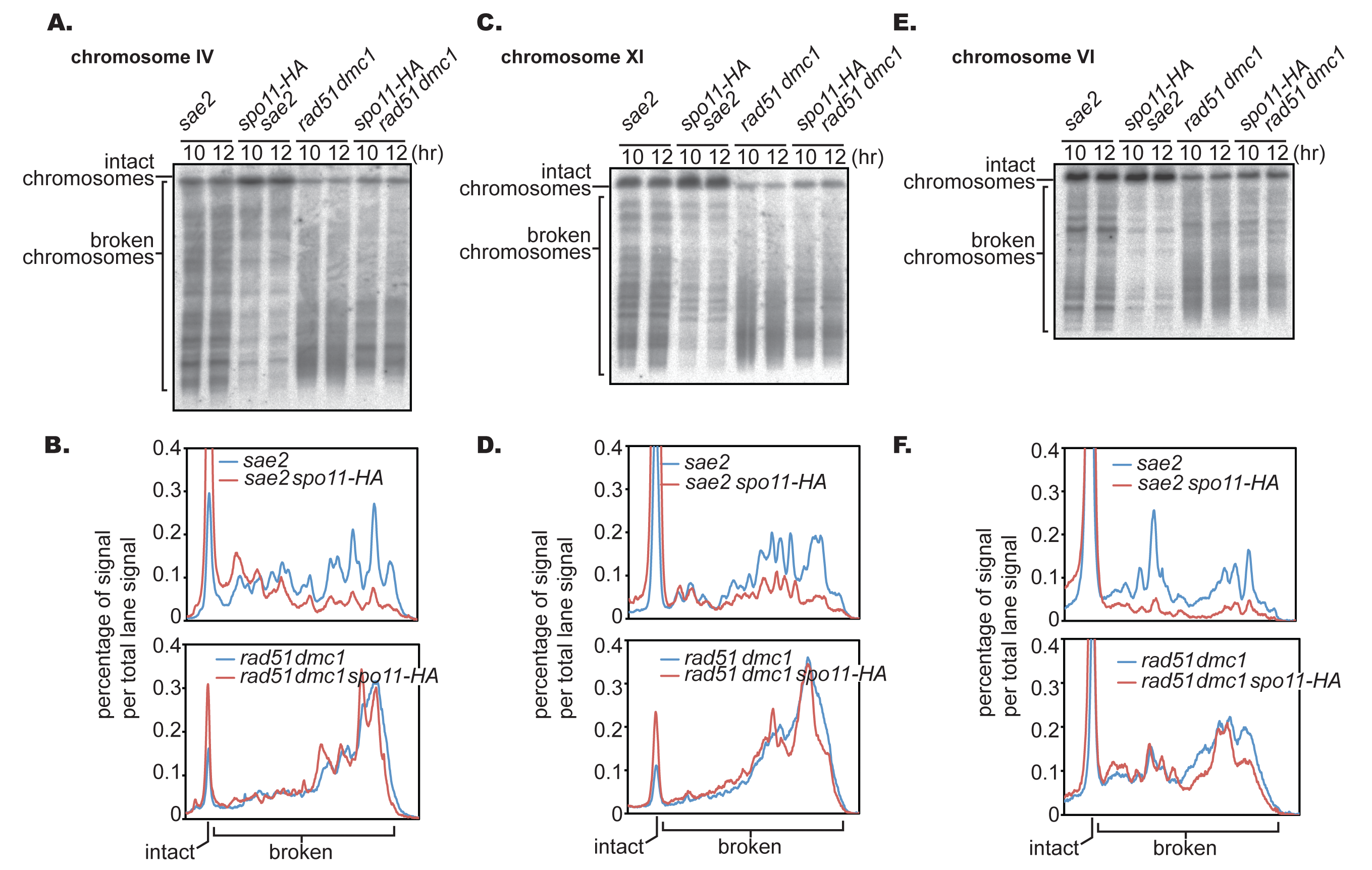

Supplement: Figure S2 — The effect of the pch2 mutation on DSB formation is different from that of spo11-HA . Experiments are done in pairs (i.e., sae2 and sae2 pch2, and sae2 and sae2 spo11-HA). Southern blot images of accumulated broken chromosomes along with normalized lane profiles are shown. Chromosome III data of sae2 and sae2 pch2 are the same as the one used in Figure 4E. sae2 and sae2 spo11-HA data are the same as part of those presented in Figure 4 and S3. (TIF) [file pone.0039724.s002.tif]

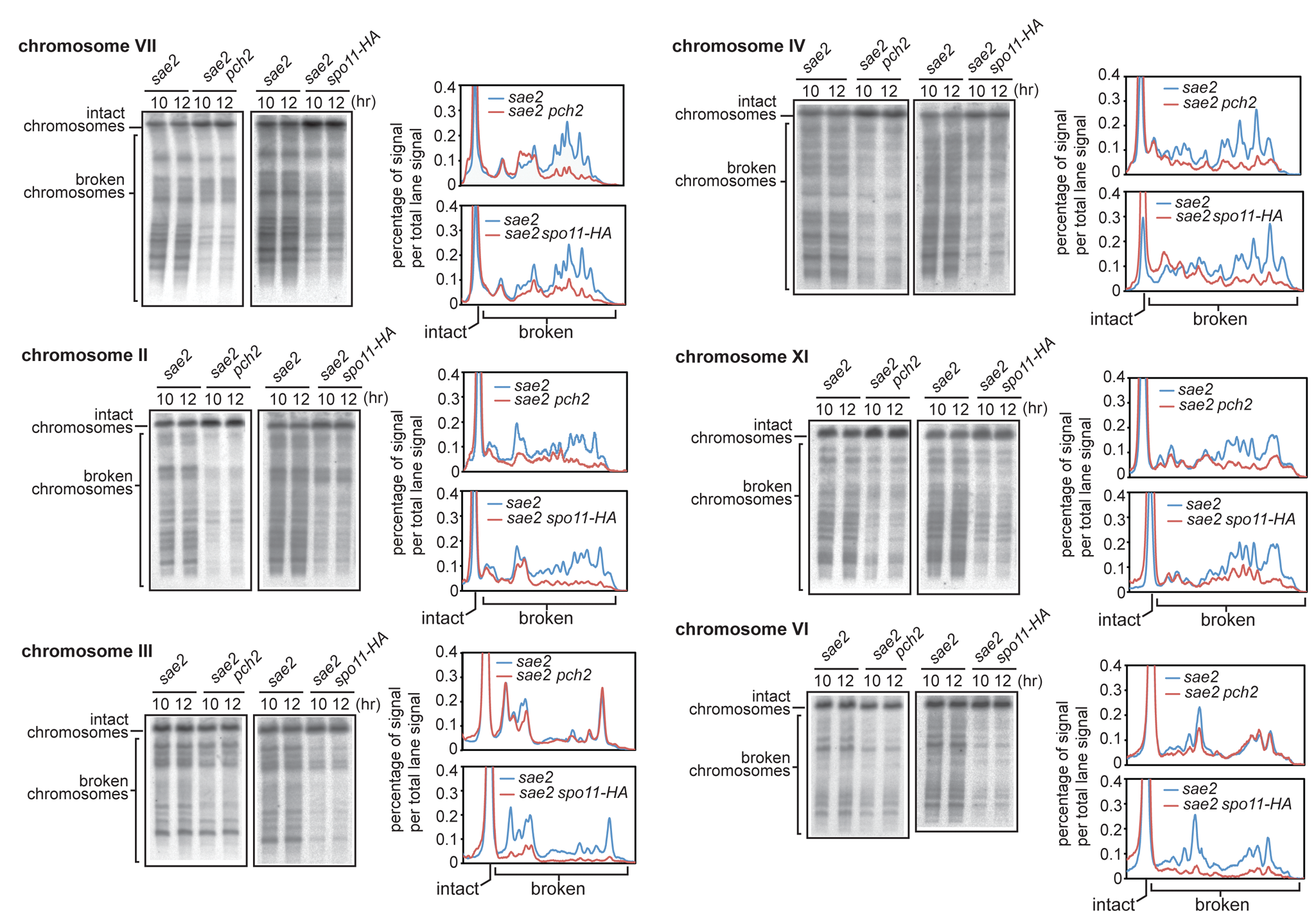

Supplement: Figure S3 — spo11-HA effect on DSB formation is more pronounced in sae2 than in rad51 dmc1. (A, C, E) Southern blot images of accumulated broken chromosomes. (B, D, F) Lane profiles of the Southern blots above. Lane profiles of 10 and 12 hours in each mutant background were normalized and averaged to obtain the profiles shown. At least two experiments were done for each genotype and a representative result is shown here. Cells from the same time course were used to examine these chromosomes and those in Figure 4. (TIF) [file pone.0039724.s003.tif]
