# Supplementary material for: Budding Yeast Pch2, a Widely Conserved Meiotic Protein, Is Involved in the Initiation of Meiotic Recombination
Source: PLoS One. 2012 Jun 22;7(6):e39724. doi: 10.1371/journal.pone.0039724 (PMC3382142; doi:10.1371/journal.pone.0039724)
Supplement: Table S1 — Strains used in this work. (PDF) [file pone.0039724.s004.pdf]

Table S1 Strains used in this work.

Strain	Genotypes
NHY187	*MAT**a** ho::hisG leu2::hisG ura3(ΔSma-Pst) HIS4::LEU2-(NBam)*
NHY285	*MATα ho::hisG leu2::hisG ura3(ΔSma-Pst) his4X::LEU2-(NBam)-URA3*
TBR5190	NHY187xNHY285
TBR5514	TBR5190 but homozygous *sae2::KAN*
TBR5515	TBR5190 but homozygous *sae2::KAN pch2::HYG*
TBR5461	TBR5190 but homozygous *dmc1::KAN*
TBR5462	TBR5190 but homozygous *dmc1::KAN pch2::HYG*
TBR5520	TBR5190 but homozygous *rad51::NAT dmc1::KAN*
TBR4664	TBR5190 but homozygous *rad51::NAT dmc1::KAN pch2::HYG*
TBR5952	TBR5190 but homozygous *sae2::KAN spo11-HA-KAN*
TBR5954	TBR5190 but homozygous *rad51::NAT dmc1::KAN spo11-HA-KAN*
TBR5520	TBR5190 but homozygous *rad51::NAT dmc1::KAN*
TBR4664	TBR5190 but homozygous *rad51::NAT dmc1::KAN pch2::HYG*
TBR3451	*MAT**a** ho::LYS2 lys2 ura3 leu2::hisG trp1::hisG*
TBR3452	*MATα ho::LYS2 lys2 ura3 leu2::hisG trp1::hisG*
TBR6621	TBR3451xTBR3452
TBR6192	TBR6621 but homozygous *rad51::URA3 dmc1::KAN ndt80::LEU2*
TBR6194	TBR6621 but homozygous *rad51::URA3 dmc1::KAN ndt80::LEU2 pch2::HYG*
TBR6396	TBR6621 but homozygous *rad51::URA3 dmc1::KAN ndt80::LEU2 spo11-HA-KAN*
TBR6397	TBR6621 but homozygous *rad51::URA3 dmc1::KAN ndt80::LEU2 pch2::HYG spo11-HA-KAN*
TBR6618	TBR6621 but homozygous *sae2::KAN ndt80::LEU2*
TBR6619	TBR6621 but homozygous *sae2::KAN pch2::HYG ndt80::LEU2*

All yeast strains are isogenic derivatives of SK1.
