# Supplementary material for: Budding Yeast Pch2, a Widely Conserved Meiotic Protein, Is Involved in the Initiation of Meiotic Recombination
Source: PLoS One. 2012 Jun 22;7(6):e39724. doi: 10.1371/journal.pone.0039724 (PMC3382142; doi:10.1371/journal.pone.0039724)
Supplement: Text S1 — Estimating DSB number in chromosomes. (PDF) [file pone.0039724.s005.pdf]

Estimating DSB number in chromosomes

Let N be the number of DSBs on a chromosome of the size T. Checking the break positions from one end of the chromosome, let X_n be the size of n-th fragment. Particularly, X_1 represents the size of DNA fragments sharing one end of the chromosome. All fragments but X_1 are not observable in the experiments. Thus, we must deduce $E[N]$, the expected value of N, from this partial observation.

When breaks occur at random uniformly on a chromosome, the breaks form a Poisson process with its break rate λ, and the inter-breaks $(X_n)_{n=1,2,\ldots,N}$ are independent and identically distributed with the exponential distribution:

(1) $$F(x) = P\{X_n \leq x\} = 1 - e^{-\lambda x},$$

and in particular, $E[X_n] = E[X_1] = 1/\lambda$. Once we obtain λ, it is easy to estimate $E[N] = \lambda T$. To determine the break rate λ, we can check the hazard rate function $h(x)$ of X_1, which is defined by

(2) $$h(x)dx = P\{x < X_1 \leq x + dx | X \geq x\} = \frac{f(x)dx}{1 - F(x)},$$

where $f(x) = dF(x)/dx$ is the density of the random variable X_1. Note that, in general (not only exponential distributions), the probability distribution function can be recovered by the corresponding hazard rate function by using the formula:

(3) $$F(x) = 1 - e^{-\int_0^x h(s)ds}.$$

When the DSBs forms a Poisson process, the hazard rate function is constant and equal to λ, which can be easily checked by using (1).

However, since real DSBs do not occur completely random and the observed hazard rate functions may not appear to be constant, it is not so straight forward to carry on the inference. Let $N(t)$ be the number of breaks in the region $[0, t]$, and $\lambda(t)$ be the intensity of DSBs at the position t defined by

(4) $$\lambda(t) = \frac{dE[N(t)]}{dt}.$$

Using the intensity $\lambda(t)$, we can estimate $E[N]$ by

(5) $$E[N] = E\left[\int_0^T \frac{dN(t)}{dt}dt\right] = \int_0^T \frac{dE[N(t)]}{dt}dt = \int_0^T \lambda(t)dt.$$

By assuming that the average of intensity is well approximated by the one of hazard rate function, i.e.,

(6) $$\int_0^T \lambda(t)dt \approx \int_0^T h(t)dt,$$

the average the number of DSBs $E[N]$ is estimated by

(7)
$$E[N] \approx \int_0^T h(t)dt = -\log(1 - F(T)) \approx -\log\left(\text{the ratio of unbroken chromosomes}\right),$$

where we use (3) in third equality. In a case when DSBs forms a Poisson process with the rate λ, (7) holds with equality. Note that, in general, (7) tends to underestimate $E[N]$ because it neglects the contribution to estimate $\lambda(t)$ from multiple breaks on a chromosome.
